# Long-Term Results of a Web-Based Guided Self-Help Intervention for Employees With Depressive Symptoms: Randomized Controlled Trial

**DOI:** 10.2196/jmir.3539

**Published:** 2014-07-09

**Authors:** Anna S Geraedts, Annet M Kleiboer, Jos Twisk, Noortje M Wiezer, Willem van Mechelen, Pim Cuijpers

**Affiliations:** ^1^Department of Clinical PsychologyVrije University AmsterdamAmsterdamNetherlands; ^2^EMGO Institute for Health and Care ResearchVU University Amsterdam and VU University Medical Center AmsterdamAmsterdamNetherlands; ^3^Body@Work, Research Center Physical Activity, Work and HealthTNO-VU-VUmcAmsterdamNetherlands; ^4^Department of Epidemiology and BiostatisticsVU University Medical CenterAmsterdamNetherlands; ^5^Department of Health Sciences, Section Methodology and Applied BiostatisticsVrije University AmsterdamAmsterdamNetherlands; ^6^TNOHoofddorpNetherlands; ^7^Department of Public and Occupational HealthVU University Medical CenterAmsterdamNetherlands

**Keywords:** depression, employees, occupational intervention, self-help, prevention, burnout, Internet

## Abstract

**Background:**

Depressive disorders are highly prevalent in the working population and are associated with excessive costs. The evidence for effective worker-directed interventions for employees with depressive symptoms is limited. Treating employees with depressive symptoms via the Internet before they report sick from work could be beneficial and cost saving.

**Objective:**

In this study, we tested the effectiveness over the period of 1 year of a Web-based guided self-help intervention, called Happy@Work, for employees with depressive symptoms who were not on sick leave.

**Methods:**

A two-arm randomized controlled trial comparing a worker-directed, Web-based, guided self-help intervention to care as usual (CAU) was carried out. We recruited employees from 6 companies via the company’s Intranet and by putting up posters. The inclusion criteria were elevated depressive symptoms as measured by a score ≥16 on the Center for Epidemiologic Studies Depression scale (CES-D) and not being on sick leave. The intervention contained 6 lessons and consisted of problem-solving treatment and cognitive therapy. Participants were asked to submit weekly assignments via the website after completion of a lesson and they received feedback from a coach via the website. Self-report questionnaires on depressive symptoms (CES-D; primary outcome), burnout (Maslach Burnout Inventory, MBI), work performance (Health and Work Performance Questionnaire, HPQ), duration of absenteeism, and anxiety (Hospital Anxiety and Depression Scale, HADS; secondary outcomes), were completed at baseline, posttreatment, and at 6-, and 12-month follow-up. Several subgroup and per-protocol analyses were performed.

**Results:**

A total of 231 employees were randomized to either the intervention group (n=116) or to CAU (n=115). Completion of assessments varied between 54%-74%. Improvement in depressive symptoms between baseline and posttreatment was shown in all participants and these effects sustained over time. However, there were no differences between the 2 groups (adjusted regression coefficient=0.46, 95% CI –2.11 to 3.03, *P*=.72; Cohen’s *d*=0.05). Differences between groups were also not significant for the secondary outcomes. No subgroups were identified to show differences between the groups, nor did we find a between-group effect in the per-protocol analyses.

**Conclusions:**

This study showed that a worker-directed, Web-based, guided self-help intervention was not more effective than CAU in reducing depressive symptoms among employees with depressive symptoms who were not on sick leave over the period of 1 year. An intervention for this specific target group might not be necessary because the recovery in the CAU group was comparable to the intervention group and sustained over a 12-month period.

**Trial Registration:**

Nederlands Trial Register (NTR): NTR2993; http://www.trialregister.nl/trialreg/admin/rctview.asp?TC=2993 (Archived by WebCite at http://www.webcitation.org/6PL9pFC0n).

## Introduction

Depressive disorders are highly prevalent in the general [1-3] and working [4,5] populations and lead to excessive costs [6,7]. Approximately 70%-85% of the costs are because of work absenteeism, work impairment, and loss of work productivity, which suggests that companies pay the largest part of the total costs of depression [8-12].

Research on the treatment of depression has been extensive and has shown that depression can be treated effectively with different forms of psychotherapies [13-18]. Traditionally, most types of psychotherapies are delivered face-to-face in mental health care settings, but there is increasing evidence for the effectiveness of treatments that are delivered via the Internet [19-23]. In general, studies on the effectiveness of Web-based interventions for the treatment of depressive symptoms show positive short-term effects [21], but there are fewer studies available that have also studied the long-term effects of Web-based interventions [21]. In a recently published meta-analysis on the effects of computer cognitive behavior therapy (CCBT) for depression, Richards and Richardson [21] reported the results of 14 studies that included a long-term follow-up, primarily up to 6 months with few studies reporting outcomes up to 12 months. They showed a small but significant effect of CCBT on depression (*d*=0.20) but stressed that more studies are needed to confirm the benefits of Web-based interventions at long-term follow-up [21].

The large number of studies on the treatment of depression in mental health care is in contrast with the few studies on worker-directed interventions for employees with depression or depressive symptoms. It is, however, important to develop evidence-based worker-directed interventions for employees with depression that involve work-related aspects, such as high job demands and work-life balance, because work-related aspects play an important role in the development and perpetuation of depression [24-26]. The Organisation for Economic Co-operation and Development (OECD) [4] has recently recommended to increase the evidence for worker-directed treatments of mental health problems and have highlighted the importance of intervening before employees take sick leave. Early intervention (before sick leave) is important because it may prevent worsening of mental health problems; consequently, it has the potential to reduce the costs of work absenteeism and loss of work productivity [4,26,27].

Several studies have been published on the effectiveness of face-to-face or Web-based worker-directed interventions for non-sick-listed employees [28-38]. Most of these studies were aimed at employees with stress or burnout symptoms who had not (yet) reported sick from work. All these studies showed positive effects of the interventions on symptom reduction. Care-as-usual (CAU) and waiting-list control groups were used most frequently as reference groups and the highest effects were seen in studies with a waiting-list control group. However, it is known that studies that use a waiting-list control comparator have a tendency to show stronger effect sizes of the intervention because they are less likely to positively affect the outcome compared with active control groups, such as CAU [39]. Two of these studies [30,31] examined face-to-face interventions for non-sick-listed employees with depressive symptoms. To our knowledge, no studies have been published on the effectiveness of Web-based worker-directed interventions for employees with depressive symptoms who are not on sick leave. Web-based treatments may be of special benefit to the working population because the employee will not have to take time off from work for therapist visits and participation in Web-based treatments is more anonymous compared to face-to-face treatment.

Considering the importance of developing Web-based worker-directed interventions for employees with depression and the limited knowledge on the long-term effects of such interventions, we conducted a randomized controlled trial with a long-term follow-up period of 12 months in which we examined the effects of such an intervention for employees with depressive symptoms who were not on sick leave compared to a CAU control group. The design of this study has been published elsewhere [40]. A process evaluation of this study (submitted paper) revealed that the intervention was conducted according to protocol and seemed feasible for further implementation. The posttreatment effects of the Web-based guided self-help intervention showed significant but small effect sizes in favor of the intervention group for anxiety symptoms and emotional exhaustion. The intervention group improved substantially on the primary outcome of depressive symptoms, but the CAU control group improved considerably as well and there was no significant difference between both groups [41]. It is of importance to examine whether the improvement in both groups is sustainable over time or if there will be an increase of depressive symptoms in 1 or both groups. Therefore, in this study we examined between-group differences over a 1-year follow-up period on depressive symptoms, burnout symptoms, work performance, and anxiety symptoms. In addition, we studied the effects of the intervention on absenteeism and we performed several subgroup analyses regarding educational level, age, gender, working hours, and baseline depression score because different effects for these subgroups might be possible.

## Methods

### Participants

**Figure 1:**
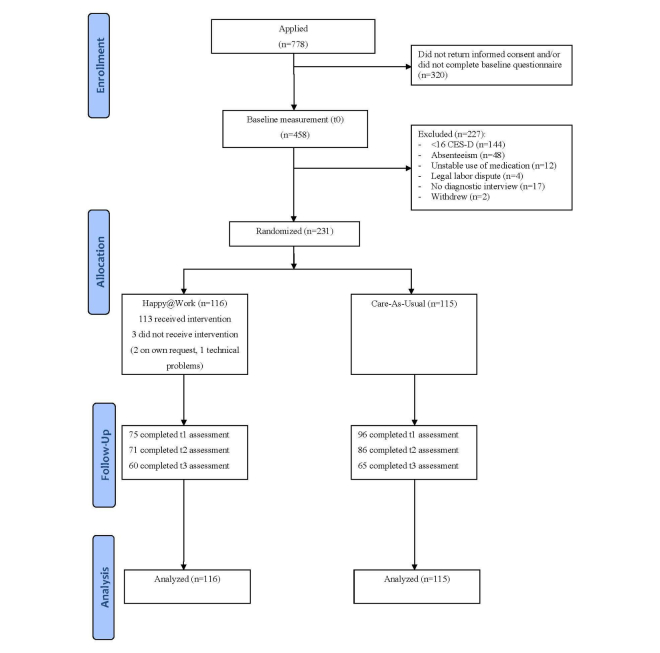
Flowchart of participants.

The design and short-term outcomes of this study have been described in detail elsewhere [40,41]. Therefore, we will describe the design briefly. Participants were recruited via 6 different companies in the Netherlands—2 banking companies, 2 research institutes, 1 security company, and 1 university—through banners and digital pamphlets on the company’s Intranet and via posters. Employees who showed interest in the study received an information leaflet and an informed consent form via email. After participants gave informed consent, they received a link to an online screening questionnaire via email. Employees with elevated depressive symptoms as measured by a score of 16 or higher on the Center for Epidemiologic Studies Depression scale (CES-D) who were not on sick leave (at the time they completed the baseline questionnaire) were eligible to take part in the study. Furthermore, access to the Internet and an email address were required. Participants were excluded if they had been using medication for depressive symptoms for less than 1 month or if they had a legal labor dispute with the employer. Once included, participants were randomized to the Web-based intervention or the CAU control group. The recruitment and retention details are shown in Figure 1.

### Procedure

This study was a randomized controlled trial with 2 arms: a Web-based guided self-help intervention (called Happy@Work) and a CAU group. The study was approved by the Medical Ethics Committee of the VU University Medical Center (registration number 2011/2) and registered in the Dutch Trial Register (NTR2993). The sample size was determined at 200 participants, based on a power of .80, an alpha of .05, and an expected dropout percentage of 30% to show a posttreatment effect size Cohen’s *d* of 0.50. A total of 231 participants were randomized to the Happy@Work intervention (n=116) or the CAU group (n=115). Randomization took place at an individual level after completion of the baseline measurement (questionnaire and clinical interview). We used stratification at 2 levels: (1) use of antidepressants and (2) receiving treatment from a psychologist or psychiatrist at study entry. Block randomization was used with random blocks containing 4, 6, or 8 allocations. An independent researcher made the allocation schedule with a computerized random number generator and the investigators had no knowledge of the schedule. The participants were informed about randomization outcome via email. Participants completed online questionnaires at baseline and posttreatment at 8 weeks (t1), 6 months (t2), and 12 months (t3).

### Interventions

#### Happy@Work

The intervention Happy@Work [42] is a brief Web-based intervention delivered with minimal guidance. It consists of 2 evidence-based treatments; problem-solving treatment (PST) [43] and cognitive therapy (CT) [44], and a guideline for employees to help them to prevent work-related stress [45,46]. Happy@Work consists of 6 weekly lessons with an option of 1 week extra time in case of delay. Each lesson has a different theme, but always follows the same structure: information about the theme, examples, and assignments. Themes of the lessons are introduction of problem solving (lesson 1), problem-solving methods (lesson 2), changing cognitions (lesson 3), dealing with work-related problems (lesson 4), social support (lesson 5), and relapse prevention (lesson 6). Participants receive feedback on assignments from a coach. Coaches were trained Master’s-level students in clinical psychology. All coaches used a protocol-treatment manual. To ensure treatment fidelity, all feedback was reviewed by a supervisor (AG) before it was placed on the website. Happy@Work is a tunneled intervention, which means that participants can start with a new lesson after they have received feedback on their assignments from a coach. Participants were viewed as treatment completers if they had followed at least the basic information and assignments of PST and CT (completion of lessons 1-3).

**Figure 2:**
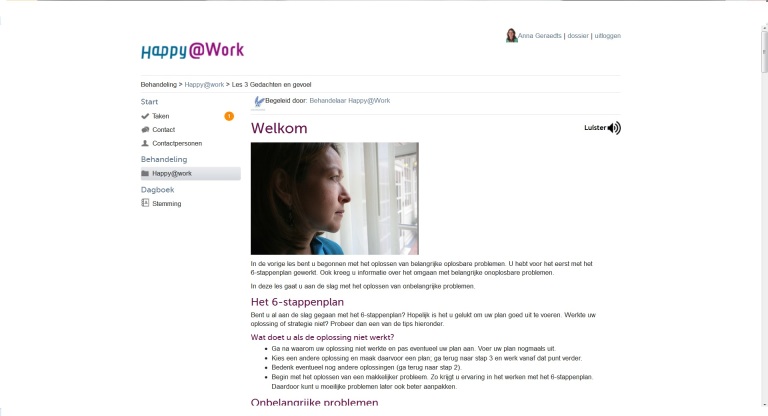
Screenshot of the Happy@Work intervention.

At the start of the intervention, an account was generated by the researchers on the website and a coach was assigned to the participant on the website. Once the account was generated, an automatic email was sent to the participant with a link to activate the account. Participants used their email address and a self-created password to log in once the account was activated. Reminders were sent to participants via email when deadlines were not met. There were no changes to the content, bugs, or periods with downtime during the trial. Screenshots of the intervention can be found in Figure 2 and in Multimedia Appendix 1.

#### Care as Usual

Participants randomized to the CAU group received an email with the randomization outcome only and were advised to consult their (occupational) physician or a psychologist if they wanted treatment for their depressive symptoms. Participants in both conditions were free to seek any additional (mental) health care.

### Measures

#### Depressive Symptoms

The primary outcome was depressive symptoms as measured by the CES-D [47]. This questionnaire is widely used for identifying people with depressive symptoms. Its validity has been tested in different populations [48-50]. The CES-D consists of 20 items and the total score varies between 0 and 60. The baseline Cronbach alpha in this study was .82. A score of 16 or higher represents a clinically significant level of depressive symptoms [47]. The cut-off score of 16 was used in this study as an inclusion criterion. This cut-off score is used frequently in studies and has shown to have good sensitivity (0.95), specificity (0.85), and positive predictive value of major depression (0.11) in a sample of employees [50].

#### Burnout Symptoms

Burnout symptoms were measured with the Dutch version of the Maslach Burnout Inventory-General Scale (MBI) [51,52]. This self-report questionnaire contains 15 items and 3 dimensions: emotional exhaustion (5 items), cynicism (4 items), and reduced professional efficacy (6 items). Every item was scored on a 7-point Likert scale (0-6). Following the manual of the questionnaire [52], a total score for every dimension was calculated by adding the item scores and by dividing the total score by the number of items, with higher scores indicating more severe symptoms. We rescored the professional efficacy dimension with higher scores indicating less feeling of professional efficacy. The baseline Cronbach alphas for the different dimensions in this study were .83 for exhaustion, .83 for cynicism, and .79 for reduced professional efficacy.

#### Work Absenteeism

Work absenteeism was measured with the second part of the Trimbos and iMTA Questionnaire on Costs Associated with Psychiatric Illness (TiC-P) the Short Form Health and Labor Questionnaire (SF-HLQ) [53]. The participant was asked to report the total number of days absent from work because of illness in the time period between the assessments at 8 weeks (t0-t1), 4 months (t1-t2), and 6 months (t2-t3). The recall period at baseline assessment was 3 months. Research has shown that participants can report valid and accurate rates of work absenteeism up to 6 months [54].

#### Work Performance

We used the general work performance scale of the World Health Organization (WHO) Health and Work Performance Questionnaire (HPQ) [55], which contains 4 items. Item 4 gives the best and easiest indication of the participant’s perception of their own work performance [56] by asking participants to rate their overall work performance during the past 4 weeks compared to employees in comparable functions. We only report on that item in this study. Work performance was scored on a 7-point Likert scale with a higher score indicating poorer work performance compared to other employees [56].

#### Anxiety Symptoms

The anxiety subscale of the Hospital Anxiety and Depression Scale (HADS) was used to measure anxiety symptoms [57]. The anxiety subscale of the HADS consists of 7 items. Scores range from 0 to 21, with higher scores indicating more anxiety. The HADS has shown good homogeneity and reliability in different normal and clinical Dutch samples [58]. The baseline Cronbach alpha in this study was .76.

#### Clinical Interview

The WHO Composite International Diagnostic Interview version 2.1 (CIDI) [59] is a structured interview to assess psychiatric diagnosis defined in the American Psychiatric Association’s *Diagnostic and Statistical Manual of Mental Disorders*, 4th edition, Text Revision (*DSM-IV-TR*) [60]. For this study, 2 sections of the CIDI were assessed: the mood disorders section and the “other” anxiety disorders (social phobia, panic disorder, agoraphobia, and generalized anxiety disorder) section. The CIDI was conducted by trained interviewers via telephone at baseline (T0) and 6-month follow-up and was used for diagnostic purposes.

#### Health Care Utilization

A revised version of the Trimbos and iMTA Questionnaire on Costs Associated with Psychiatric Illness (TiC-P) [53] was used to collect data on health care utilization. The TiC-P is a self-report questionnaire and consists of 2 different parts that can be administrated separately. Part I was used, which contains 12 items concerning health care utilization by participants. There were 2 questions added to the questionnaire about the frequency of utilization of different health care services of the company: occupational physician and occupational social work. The questionnaire was used at T0 to assess health care utilization up to 3 months before the start of the study and at posttreatment (t1) assessment to assess health care utilization between baseline and posttreatment assessment.

#### Other Measures

We included several demographic questions and questions about working hours and working days in the baseline questionnaire.

### Statistical Analyses

#### Effectiveness

Linear mixed modeling (LMM) was used to examine treatment differences. Two LMM analyses were performed: (1) unadjusted analyses, only controlling for the baseline score of the outcome measure and (2) adjusted analyses, controlling for other baseline variables, such as age, gender, marital status, educational level, nationality, and working hours, as well as the baseline outcome score. In LMM analyses, the regression coefficient represents the overall mean difference between the groups over time, so over all assessments after baseline. Reporting the overall mean difference between the groups over time was chosen because we were interested in the difference between the groups over the entire period of 1 year. If the regression coefficient is positive, the mean difference is in favor of the intervention group; if the regression coefficient is negative, the mean difference is in favor of the CAU group.

An overall between-group effect size for every outcome variable was calculated according to Cohen’s *d* [61]. The Cohen’s *d* was calculated by dividing the overall mean difference between the groups (expressed as regression coefficient) by the overall SD of the observed data. Effect sizes ≥0.8 are assumed to be large, effect sizes between 0.5-0.8 are moderate, and effect sizes between 0.2-0.5 are assumed to be small [61]. Furthermore, in additional analyses we calculated the Cohen’s *d* for depressive symptoms on every assessment based on the observed data. The Cohen’s *d* was calculated by subtracting the mean score of the CAU group from the mean score of the intervention group and dividing that result by the pooled standard deviation.

All analyses were performed according to the intention-to-treat (ITT) principle. Missing data were handled by multiple imputation via data augmentation. Data augmentation is an iterative Markov chain Monte Carlo method to generate the imputed values assuming a multivariate normal distribution. Five imputations were used in all analyses and reported in the effectiveness analyses. Results of the mean and standard deviations reported are of the observed data.

#### Subgroup and Per-Protocol Analyses

We performed several a priori subgroup analyses on the primary outcome depressive symptoms. These subgroup analyses were educational level, age (age <35 versus age ≥35), gender, working full time (≥36 hours per week) versus part time (<36 hours per week), and high baseline score as defined by a score of ≥27 on the CES-D (used more often as an indicator of more severe depressive symptoms) [62-64]. In the subgroup analyses, the specific subgroup was selected from all study participants and the difference between the groups over time was then compared.

Furthermore, we performed a per-protocol analysis based on treatment completers (completed ≥3 lessons of the intervention).

#### Sensitivity Analyses

We also performed all analyses on the data for 100 imputations. Because there is a current debate whether it is necessary to perform multiple imputations in combination with mixed-model analyses in longitudinal studies [65], we also performed the LMM analyses without multiple imputations. All multiple imputations and LMM analyses were performed in STATA version 11.2 (StataCorp LP, College Station, TX, USA) with the procedures mi and xtmixed.

## Results

### Participants and Response Rates

Figure 1 shows the flow of participants through the trial. A total of 231 participants were included in the trial, 29.7% (231/778) of the employees who showed initial interest in the study. Of these, 116 participants were randomized to the intervention group and 115 to the CAU group. Most participants (n=166) were employed by 1 of the 2 banking companies, 39 by the 2 research institutes, 11 by the security company, and 15 by the university. Of the 231 participants, 10 (4.3%) used medication without psychological treatment, 24 (10.4%) received psychological treatment but no medication, and 4 participants (1.7%) used both medication and received psychological treatment at baseline. Thus, most participants in both groups (83.6%, 193/231) were not receiving treatment for their depressive symptoms at baseline.

As shown in Table 1, most participants were female (62.3%, 144/231), born in the Netherlands (95.2%, 220/231), involved in an intimate relationship (76.2%, 176/231), highly educated (63.6%, 147/231), and worked for 34 hours per week on average.

**Table 1:** Participants’ demographic characteristics at baseline.

Characteristic		All (N=231)	Intervention (n=116)	CAU (n=115)	*P*
Age (years), mean (SD)		43.4 (9.2)	43 (8.9)	43.8 (9.6)	.51
**Gender, n (%)**					.20
	Female	144 (62.3)	77 (66.4)	67 (58.3)	
	Male	87 (37.7)	39 (33.6)	48 (41.7)	
**Country of birth, n (%)**					.03
	Netherlands	220 (95.2)	107 (92.2)	113 (98.3)	
	Other	11 (4.8)	9 (7.8)	2 (1.7)	
**Marital status, n (%)**					.46
	Relationship	176 (76.2)	86 (74.1)	90 (78.3)	
	No relationship	55 (23.8)	30 (25.9)	25 (21.7)	
**Education,** ^a^ **n (%)**					.25
	Low	16 (6.9)	11 (9.5)	5 (4.3)	
	Middle	68 (29.4)	31 (26.7)	37 (32.2)	
	High	147 (63.6)	74 (63.8)	73 (63.5)	
Working hours,^b^ mean (SD)		33.9 (5.0)	33.7 (4.8)	34.0 (5.3)	.65
Working days, mean (SD)		4.3 (0.7)	4.3 (0.6)	4.2 (0.7)	.32

^a^Low: primary education or lower general secondary education; middle: intermediate vocational education or high school; high: higher vocational education or university.

^b^Mean working hours per week according to contract of the employee.

### Diagnosis

All participants completed the baseline clinical interview. At 6-month follow-up, a total of 170 participants (73.6%, 170/231) participated in the clinical interview. A total of 57 participants (24.7%) were diagnosed with a current major depressive disorder, dysthymic disorder, or both at baseline: 23 participants from the intervention group and 34 in the CAU group. At 6-month follow-up, 19 participants were diagnosed with a current major depressive disorder, dysthymic disorder, or both: 6 participants from the intervention group and 13 in the CAU group. From the 57 participants who were diagnosed with a current major depressive disorder, dysthymic disorder, or both at baseline, 9 participants suffered from a current major depressive disorder, dysthymic disorder, or both at 6-month follow-up as well: 2 participants from the intervention group and 7 participants from the CAU group. There were 10 participants who were diagnosed with a current major depressive disorder, dysthymic disorder, or both at 6-month follow-up but not at baseline. Of those 10 participants, 4 participants were from the intervention group and 6 participants were from the CAU group.

### Health Care Utilization

At posttreatment, we analyzed the health care utilization of both groups to get a more detailed view on health care utilization by the CAU group. Only a small number of the total participants made use of health care and this was not significantly different between the groups. A detailed description of health care use can be found elsewhere [41].

### Attrition and Adherence

#### Study Attrition

The attrition rates for the study sample were 26% at posttreatment assessment, 32% at the 6-month follow-up assessment, and 46% at the 12-month follow-up assessment. Participants in the CAU group completed the posttreatment assessment (χ^2^_1_=11.5, *P*=.001) and the 6-month follow-up assessment (χ^2^_1_=4.9, *P*=.03) more often. There were no differences between the groups for completion of the 12-month follow-up assessment. Attrition rates for the posttreatment assessment were lower in participants who completed the intervention (χ^2^_1_=32.1, *P*<.001).

#### Intervention Adherence

Of the 116 participants randomized to the intervention group, 9.5% (11/116) did not start or complete the first lesson of Happy@Work. A total of 67 participants (57.8%) were seen as treatment completers because they completed 3 or more lessons of the intervention. A total of 29 of 116 participants dropped out of the intervention at their own request or because of prolonged inactivity on the website. The other participants were not able to complete more lessons within the time limit of 7 weeks. Most participants who dropped out did not report a reason for dropout (15/116, 12.9%). When reasons were reported (14/116), they pertained mostly to lack of time (8/14, 57.1%).

**Table 2:** Observed scores of the intervention and care-as-usual (CAU) groups on different outcome measures.

Outcome		Assessment time, mean (SD)			
		Baseline (t0) (n=231)	Posttreatment (t1) (n=171)	Follow-up 6 months (t2) (n=157)	Follow-up 12 months (t3) (n=125)
**CES-D**					
	Intervention	25.7 (7.5)	15.8 (10.6)	15.7 (11.3)	13.8 (9.7)
	CAU	26.1 (7.0)	18.3 (9.1)	14.5 (8.9)	16.2 (10.7)
**MBI-exhaustion**					
	Intervention	3.3 (1.2)	2.7 (1.2)	2.6 (1.4)	2.3 (1.4)
	CAU	3.3 (1.1)	3.0 (1.2)	2.5 (1.2)	2.5 (1.3)
**MBI-cynicism**					
	Intervention	2.8 (1.3)	2.4 (1.3)	2.5 (1.5)	2.4 (1.4)
	CAU	3.1 (1.3)	2.8 (1.3)	2.4 (1.3)	2.4 (1.4)
**MBI-reduced professional efficacy**					
	Intervention	2.6 (1.0)	2.4 (1.0)	2.3 (1.1)	2.2 (1.2)
	CAU	2.7 (0.9)	2.5 (0.9)	2.3 (0.9)	2.3 (1.1)
**Absenteeism (days)** ^a^					
	Intervention	1.8 (2.7)	0.4 (1.0)	3.6 (9.4)	7.3 (25.6)
	CAU	2.0 (3.3)	1.6 (4.9)	5.0 (13.7)	6.9 (23.3)
**Work performance**					
	Intervention	4.1 (1.6)	3.6 (1.5)	3.6 (1.5)	3.6 (1.5)
	CAU	4.3 (1.8)	3.6 (1.5)	3.6 (1.5)	3.7 (1.6)
**HADS**					
	Intervention	10.6 (3.8)	7.6 (3.8)	6.8 (4.1)	6.6 (4.1)
	CAU	10.2 (3.2)	8.3 (3.6)	7.2 (4.0)	6.8 (4.0)

^a^Recall periods differed per assessment: 3 months (t0), 8 weeks (t1), 4 months (t2), 6 months (t3).

### Effectiveness

All participants improved between baseline and posttreatment on the primary outcome depressive symptoms and this improvement sustained over time (see Table 2). However, the overall estimated mean difference between the groups over time was not significant (see Table 3). This indicates that the estimated mean difference between the groups over the period of 1 year was not significant. The overall between-group effect size was small (*d*=0.05). The Cohen’s *d* per assessment were all small to moderate effect sizes and nonsignificant (t1: *d*=0.26, 95% CI –0.04 to 0.56; t2: *d*=–0.12, 95% CI –0.43 to 0.20; t3: *d*=0.24 95% CI –0.12 to 0.59).

For the secondary outcomes, the same pattern of results was seen as with the depressive symptoms. There were improvements between baseline and posttreatment assessment on the secondary outcomes and these improvements sustained over time (see Table 2), but there were no significant differences between the groups over time. The overall between-group effect sizes for the secondary outcomes were all small (see Table 3). The absenteeism outcome was expressed in duration of absenteeism during the time period between 2 assessments. Therefore, it is not possible to study whether there was an increase or decrease of absenteeism duration over time, but only the differences between the groups on absenteeism duration can be examined. The overall estimated mean difference between the groups over time was not significant (see Table 3).

**Table 3:** Overall effectiveness on different outcome measures.

Outcome	Unadjusted coefficient^a^	95% CI	*P*	Effect size^b^	Adjusted coefficient^c^	95% CI	*P*	Effect size^b^
CES-D	0.14	–2.00, 2.27	.90	0.01	0.46	–2.11, 3.03	.72	0.05
MBI-exhaustion	0.10	–0.14, 0.33	.42	0.08	0.10	–0.13, 0.33	.40	0.08
MBI-cynicism	–0.08	–0.33, 0.17	.54	–0.06	–0.07	–0.32, 0.18	.57	–0.05
MBI-reduced professional efficacy	0.00	–0.24, 0.24	.98	0.00	0.04	–0.20, 0.27	.76	0.04
Absenteeism	–0.01	–4.69, 4.67	.99	0.00	–0.89	–6.09, 4.31	.72	0.04
Work performance	0.05	–0.24, 0.35	.72	0.03	0.01	–0.30, 0.32	.94	0.01
HADS	0.48	–0.29, 1.25	.22	0.12	0.60	–0.19, 1.38	.13	0.15

^a^Unadjusted regression coefficient: analyses adjusted for baseline outcome score.

^b^The effect size is presented as an overall effect size represented as Cohen’s *d*: the number of standard deviations in the intervention group has improved more than the CAU group.

^c^Adjusted regression coefficient: analyses adjusted for baseline variables and baseline outcome score.

### Subgroup Analyses

Data from the a priori subgroup analyses are reported in Table 4. There were no significant differences in depressive symptoms between the groups over time in any of the subgroups. Because the coefficients from the different subgroups were not substantially different from each other, there were no additional interaction effects tested to study whether there was a difference between the different subgroups over time.

**Table 4:** Overall effectiveness on depressive symptoms in different subgroups.

Subgroup		Unadjusted coefficient^a^	95% CI	*P*
**Gender**				
	Female	0.60	–2.13, 3.33	.66
	Male	–0.35	–4.06, 3.37	.85
**Educational level**				
	Low	–0.24	–11.95, 11.46	.97
	Middle	1.12	–3.30, 5.53	.61
	High	–0.34	–2.89, 2.21	.80
**Baseline CES-D score**				
	Score <27	0.76	–2.05, 3.60	.59
	Score ≥27	–0.37	–4.62, 3.89	.86
**Age**				
	Age <35	–0.22	–5.10, 4.66	.93
	Age ≥35	0.28	–2.05, 2.60	.82
**Working hours**				
	Work part time	–0.95	–4.05, 2.16	.55
	Work full time	0.93	–1.95, 3.82	.52

^a^Unadjusted regression coefficient: analyses adjusted for baseline depression score.

### Per-Protocol Analyses

The per-protocol analyses, in which the group of treatment completers was compared to the CAU group, did not reveal any significant results on the primary outcome depressive symptoms (unadjusted regression coefficient=–0.48, 95% CI –4.28 to 3.33, *P*=.80) and all secondary outcomes. The overall estimated mean difference for the MBI exhaustion dimension was 0.10 (95% CI –0.24 to 0.43, *P*=.57), for the MBI cynicism dimension it was 0.14 (95% CI –0.21 to 0.49, *P*=.42), for the MBI reduced professional efficacy dimension it was –0.03 (95% CI –0.48 to 0.41, *P*=.88), for work performance it was –0.14 (95% CI –0.79 to 0.51, *P*=.65), for absenteeism it was –1.66 (95% CI –7.10 to 3.78, *P*=.54), and for anxiety symptoms it was 0.08 (95% CI –1.06 to 1.23, *P*=.89).

### Sensitivity Analyses

The analyses from the datasets without imputations and with 100 imputations did not reveal any relevant differences compared to the outcomes from the dataset with 5 imputations (data not shown).

## Discussion

### Principal Results

This study examined the long-term effects of a worker-directed, Web-based, guided self-help intervention on depressive symptoms, several work-related outcome measures, and anxiety symptoms compared to CAU in employees with depressive symptoms who were not on sick leave. This study did not affirm evidence for the long-term effectiveness of the Web-based intervention compared to CAU for any of the outcome measures. Overall, participants improved substantially on the primary outcome depressive symptoms between baseline and posttreatment assessment and these improvements sustained over the period of 1 year. This was also true for the work-related outcomes of burnout symptoms and work performance as participants improved between baseline and posttreatment with sustainable effects up to 12 months. Overall, participants further improved after posttreatment assessment on anxiety symptoms. However, no difference between the 2 conditions in the course of symptoms was found on any of the outcome measures. Furthermore, there were no significant mean differences between the groups on duration of absenteeism during the follow-up period. We were not able to identify any subgroups that benefited from the treatment compared to CAU. Participants with a relatively high or low score on depressive symptoms, male or female, age <35 or >35 years, working part time or full time, having a low, middle, or high educational level, or who had completed treatment or not did not improve more than the CAU group with respect to their depressive symptoms.

### Comparison With Prior Work

The results of this study regarding depressive symptoms are not in line with the positive findings of the meta-analysis on the long-term effects of CCBT for depression by Richards and Richardson [21]. The Cohen’s *d*s that were assessed at each time point, based on the observed data, showed effect sizes that were close to the overall effect size of Richards and Richardson, but they were not significant and the effect size over time was small (*d*=0.05). There are 2 important differences between the studies analyzed in the meta-analysis and our study which make the results of the meta-analysis more difficult to compare to this study: (1) in general, the studies in the meta-analysis examined a target group with more severe depressive symptoms and/or depressive disorders at baseline compared to this study, and (2) none of the studies in the meta-analysis were tested in a workplace context. Two studies have been published on the effects of Web-based interventions in a workplace context that included long-term follow-up results and that focused on a comparable target group of non-sick-listed employees with mild to moderate depressive symptoms at baseline. Both studies tested unguided Web-based interventions. One of these interventions was a worker-directed intervention [28] and the other had no specific focus on work-related problems [66]. Both studies did not report significant effects in favor of the intervention at follow-up (either 3 or 6 months) and showed the same pattern of improvement as was found in this study: substantial improvements between baseline and posttreatment which sustained at follow-up in both groups. This pattern of improvement was also seen in this study, but not in other studies with long-term follow-up assessments [21].

The large reduction in depressive symptoms in the CAU group between baseline and posttreatment was unforeseen and sustained at the follow-up assessments [34,67,68]. We discussed several potential reasons for the large reduction of depressive symptoms in the CAU group when we reported the posttreatment effectiveness of this study. These were spontaneous recovery, a phenomenon which is seen more often in patients with depression [69], recruitment of highly motivated employees who were willing to change which could have let to improvement by itself, positive influences of work (ie, being able to function and stay at work while experiencing depressive symptoms might have had a positive influence on recovery of depressive symptoms), a company’s participation in this study gives a positive signal of an open environment to employees (ie, a change in organizational culture) which could have led to participants in the CAU group discussing their mental health problems with their supervisor which can result in reduction of depressive symptoms, and the email with randomization outcome for the CAU group contained advice to seek treatment for depressive symptoms. This email could have instigated a behavioral change according to the stages-of-change model from Prochaska and colleagues [70]. Only a small percentage of the participants in the CAU group reported having received professional help. However, it could be possible that other participants received help in a different way; for example, via their significant other or via other self-help treatments. In relation to the latter reason for the reduction of symptoms in the CAU group, it could also be possible that for this specific target group filling in a questionnaire about depression during a period of sad mood could have been enough of an intervention by itself. Considering the comparable pattern of findings of this study and the study of Grime [28] and Phillips and colleagues [66] in non-sick-listed employees, it may be possible that spontaneous recovery of depressive symptoms is more common in this specific target group, but all these reasons could have contributed to the large improvements in the control group.

When we examined the posttreatment effects of the Web-based guided self-help intervention on burnout symptoms [41], we found small but significant differences in favor of the intervention group for emotional exhaustion but not on the other 2 dimensions, cynicism and reduced professional efficacy. We explained this finding by postulating that a change in emotional exhaustion might show a first indication of treatment effect on burnout and that the other dimensions, cynicism and reduced professional efficacy, would follow because these are related to cognitions and attitudes that generally take a longer time to show improvement. Apparently, this was not the case because no further improvements on the cynicism and reduced professional efficacy dimensions occurred at follow-up, but the small improvements between baseline and posttreatment assessment sustained during follow-up.

To our knowledge, this is the first study on Web-based interventions that has used absenteeism as an outcome measure. We did not find between-group differences in absenteeism, but we were not able to investigate if there was an increase or decrease in absenteeism over time because of the use of different time periods between assessments. Future research on Web-based interventions, especially when tested in the workplace context, should include absenteeism duration and frequency as an outcome measure.

### Limitations

This study has several limitations. The first has to do with the attrition rate and handling of missing data. We were confronted with a high attrition rate which is seen more often in Web-based interventions [71,72]. The attrition rates in this study were equal or lower compared with several similar studies on guided Web-based interventions for depression with long-term follow-up assessments [67,73,74]. The bias that may have been introduced was accounted for by applying multiple imputation techniques. Because of the current debate on the necessity of multiple imputations in combination with mixed-model analysis in longitudinal studies [65], we also performed mixed-model analysis without multiple imputations. The results were comparable, indicating that data were robust and multiple imputations may not have been needed. Second, the participants in this study were primarily Dutch white-collar workers with a high educational level. Therefore, it is uncertain whether the results can be generalized to the general working population or employees with a lower education level. Although our subgroup analysis on educational level did not show significant differences, the subgroup analyses had a lack of power and only 36.4% of the study population had a low or middle educational level. Third, the power-analysis was based on a posttreatment effect and, therefore, the analyses on follow-up assessments have a lack of power. Finally, as stated previously, adherence to the intervention was low and only 57.8% completed at least 3 lessons of the intervention. Therefore, the analyses of comparisons between the intervention group and the CAU group compared the effects of a low adherence intervention, with many participants who only followed a small part of the intervention. The per-protocol analyses did not show significant differences either, but had a lack of power because the analyses were only based on 42.2% of the intervention group.

### Implications and Future Research

The results of this study implicate that the intervention Happy@Work is not more effective in reducing depressive symptoms than CAU over the period of 1 year. Overall, participants improved substantially between baseline and posttreatment assessment on depressive symptoms and these improvements sustained over time. Participants also improved on the secondary outcomes, which sustained over time. The large improvements on depressive symptoms in the CAU group were also found in 2 studies with comparable target groups of non-sick-listed employees [28,66]. Therefore, it could be possible that spontaneous recovery of depressive symptoms is more likely in this specific target group. Observational research following non-sick-listed depressed employees over time could provide more insight.

The process evaluation that was performed alongside this trial concluded that the intervention was feasible for further implementation. However, based on the results of this trial we do not recommend to directly implement Happy@Work into routine practice because it was not more effective than CAU over time. It could, however, be possible that the intervention, even though it is not effective from a clinical perspective, could be effective from an economical perspective (eg, cost-effective). This needs further investigation. Further, more research is needed to examine the possibilities of using e-mental health in the workplace setting. This research should focus on the needs of employees with mental health problems and on the ideal moment when intervention is really necessary.

### Conclusions

This study showed that the Web-based, worker-directed, guided self-help intervention Happy@Work is not more effective in reducing depressive symptoms than a CAU group over the period of 1 year. Based on the results of this study, we can conclude that an intervention for employees with mild to moderate depressive symptoms who are not on sick leave might not be necessary because the natural recovery in the CAU group was comparable to the intervention group and sustainable over a 12-month period.

## Multimedia Appendix 1

Screenshots of the intervention.

## Multimedia Appendix 2

CONSORT-EHEALTH checklist V1.6.2 [75].

## Abbreviations

CAU: care as usual
CCBT: computer cognitive behavior therapy
CES-D: Center for Epidemiological Studies Depression scale
CT: cognitive therapy
HADS: Hospital Anxiety and Depression Scale
HPQ: Health and Work Performance Questionnaire
ITT: intention-to-treat
LMM: linear mixed modeling
MBI: Maslach Burnout Inventory-General Scale
PST: problem-solving treatment
