# Supplementary material for: Long-Term Results of a Web-Based Guided Self-Help Intervention for Employees With Depressive Symptoms: Randomized Controlled Trial
Source: J Med Internet Res. 2014 Jul 9;16(7):e168. doi: 10.2196/jmir.3539 (PMC4115257; doi:10.2196/jmir.3539)
Supplement: Supplementary file 1 [file jmir_v16i7e168_app1.pdf]

Multimedia Appendix 1

Anna S. Geraedts[123]*, MSc; Annet M. Kleiboer[123]*, PhD; Jos Twisk[45]*, PhD; Noortje M. Wiezer[36]*, PhD; Willem van Mechelen[237]*, PhD, MD; Pim Cuijpers[123]*, PhD

Affiliations:

[1]Department of Clinical Psychology, VU University, Amsterdam, the Netherlands

[2]EMGO Institute for Health and Care Research, VU University Amsterdam and VU University Medical Center Amsterdam, the Netherlands

[3]Body@Work, Research Center Physical Activity, Work and Health, TNO-VU-VUmc, Amsterdam, the Netherlands

[4]Department of Epidemiology and Biostatistics, VU University Medical Center, Amsterdam, the Netherlands

[5]Department of Health Sciences, Section Methodology and Applied Biostatistics, VU University, Amsterdam, the Netherlands

[6]TNO, Hoofddorp, the Netherlands

[7]Department of Public and Occupational Health, VU University Medical Center, Amsterdam, the Netherlands

The following slides show screenshots from the participant view of the platform. Discriptions of the screenshots are given in English, but the screenshots are in Dutch.

Tasks

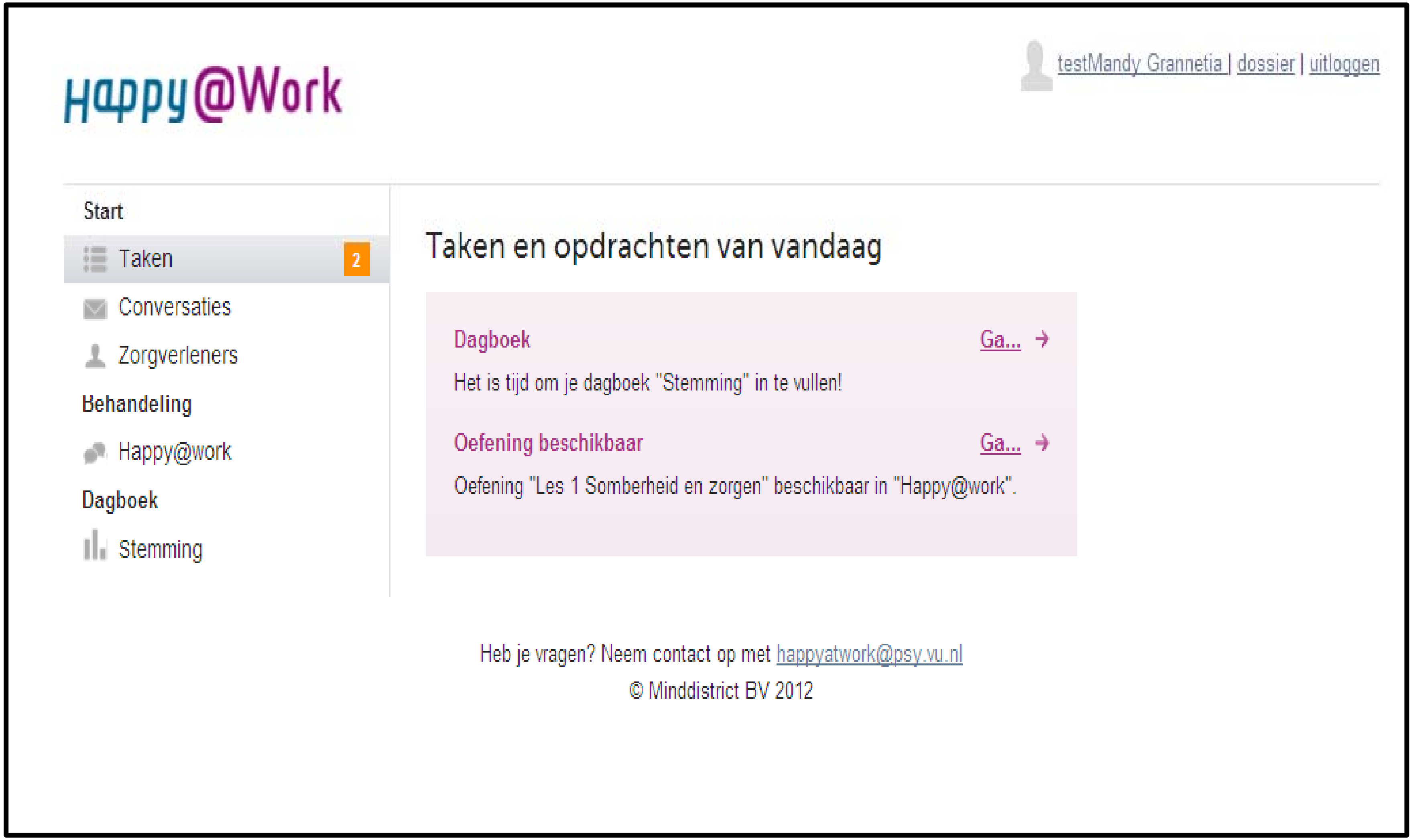
Happy@Work
testMandy Grannetia | dossier | uitloggen
Start
Taken 2
Conversaties
Zorgverleners
Behandeling
Happy@work
Dagboek
Stemming
Taken en opdrachten van vandaag
Dagboek Ga...
Het is tijd om je dagboek "Stemming" in te vullen!
Oefening beschikbaar Ga...
Oefening "Les 1 Somberheid en zorgen" beschikbaar in "Happy@work".
Heb je vragen? Neem contact op met happyatwork@psy.vu.nl
© Minddistrict BV 2012

Overview

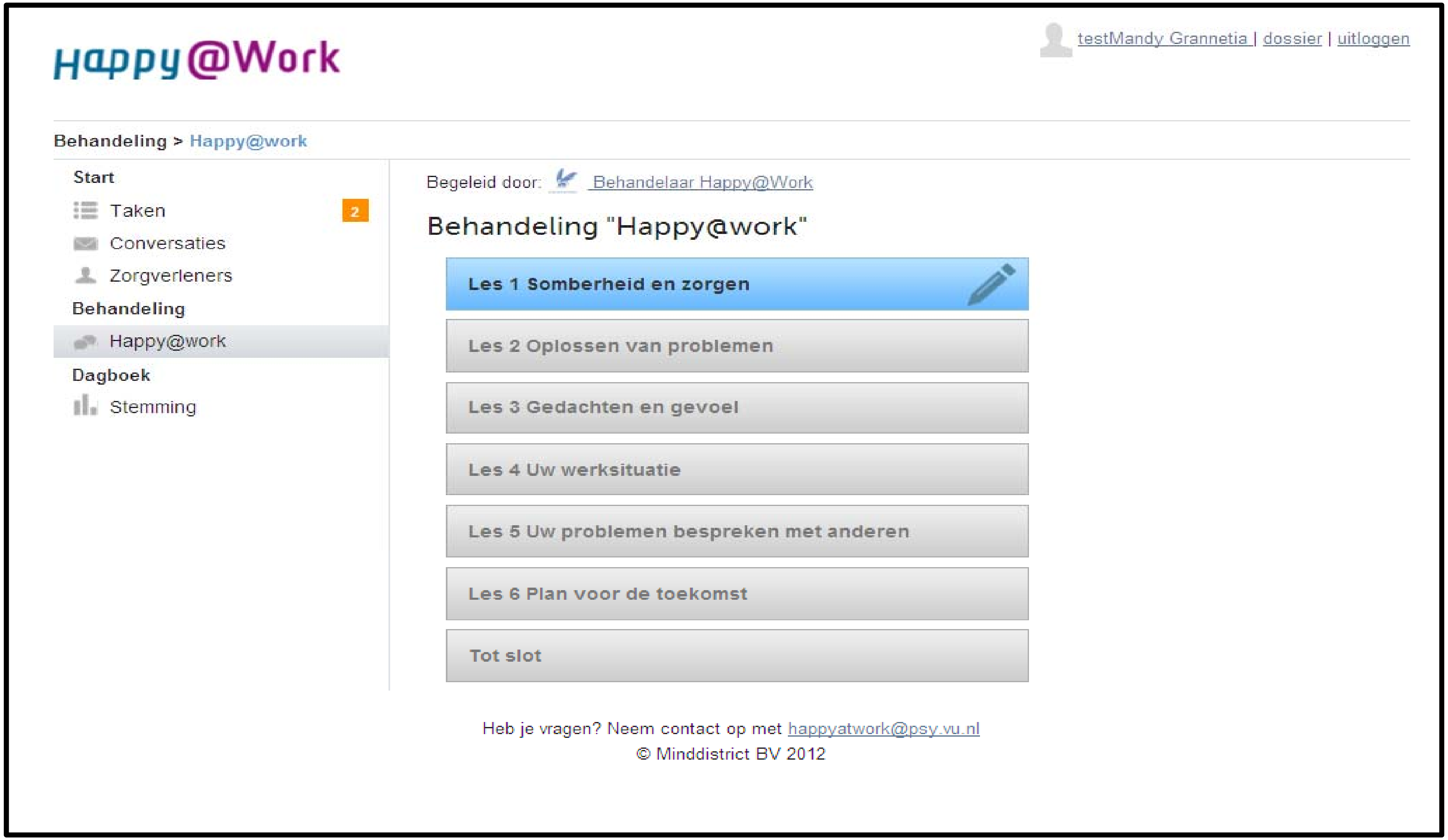
Happy@Work
testMandy Grannetia | dossier | uitloggen
Behandeling > Happy@work
Start
Taken
2
Conversaties
Zorgverleners
Behandeling
Happy@work
Dagboek
Stemming
Begeleid door: Behandelaar Happy@Work
Behandeling "Happy@work"
Les 1 Somberheid en zorgen
Les 2 Oplossen van problemen
Les 3 Gedachten en gevoel
Les 4 Uw werksituatie
Les 5 Uw problemen bespreken met anderen
Les 6 Plan voor de toekomst
Tot slot
Heb je vragen? Neem contact op met happyatwork@psy.vu.nl
© Minddistrict BV 2012

Lesson 1

Happy@Work

testMandy Grannetia | dossier | uitloggen

Behandeling > Happy@work > Les 1 Somberheid en zorgen

Start
- Taken 2
- Conversaties
- Zorgverleners

Behandeling
- Happy@work

Dagboek
- Stemming

Begeleid door: Behandelaar Happy@Work

Welkom

Luister

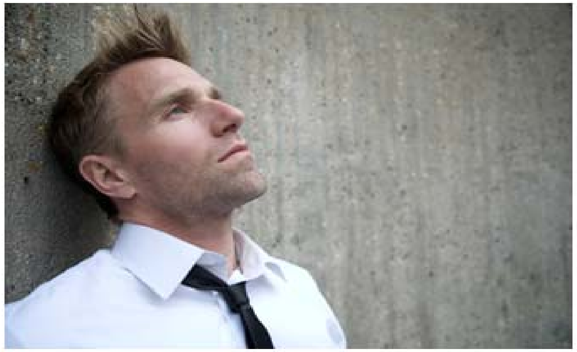

Welkom bij de cursus Happy@Work. Voordat u begint, leggen wij u graag eerst wat uit over de cursus.

Hoe werkt de cursus?

Deze cursus bestaat uit zes lessen. Elke week volgt u één les. Elke les begint met informatie. Die informatie leest u eerst helemaal door. Daarna maakt u de opdrachten bij de les. Bent u benieuwd hoe andere cursisten de opdrachten maken? Bekijk dan onder het kopje "Oefening" de voorbeeldcursisten Harmen, Suzan en Maaike.

Aan het eind van de week, als u klaar bent met uw opdrachten, stuurt u de opdrachten naar uw behandelaar. Binnen drie werkdagen krijgt u een reactie: 'feedback' noemen we dat. Zodra uw behandelaar feedback heeft gegeven, krijgt u daarvan bericht per e-mail. De feedback zelf kunt u lezen op de website. Daarna kunt u doorgaan met de volgende les.

Assignment

Problemen en zorgenlijst

Hieronder kunt u in het linkerblokje onder 'zorg/probleem' uw probleem opschrijven. Kies in het blokje daarnaast of het gaat om een onbelangrijk probleem, een belangrijk oplosbaar probleem, of een belangrijk maar niet oplosbaar probleem. Klik op het knopje '+' of "Add" om het probleem aan uw lijst toe te voegen.

Zorg/probleem	Soort probleem	
Baangarantie	Belangrijk maar niet oplosbaar	✖
Te weinig persoonlijke ontwikkeling binnen mijn baan	-<br>-<br>Belangrijk en oplosbaar<br>Onbelangrijk<br>Belangrijk maar niet oplosba	✖

✚ Toevoegen

Mood Diary

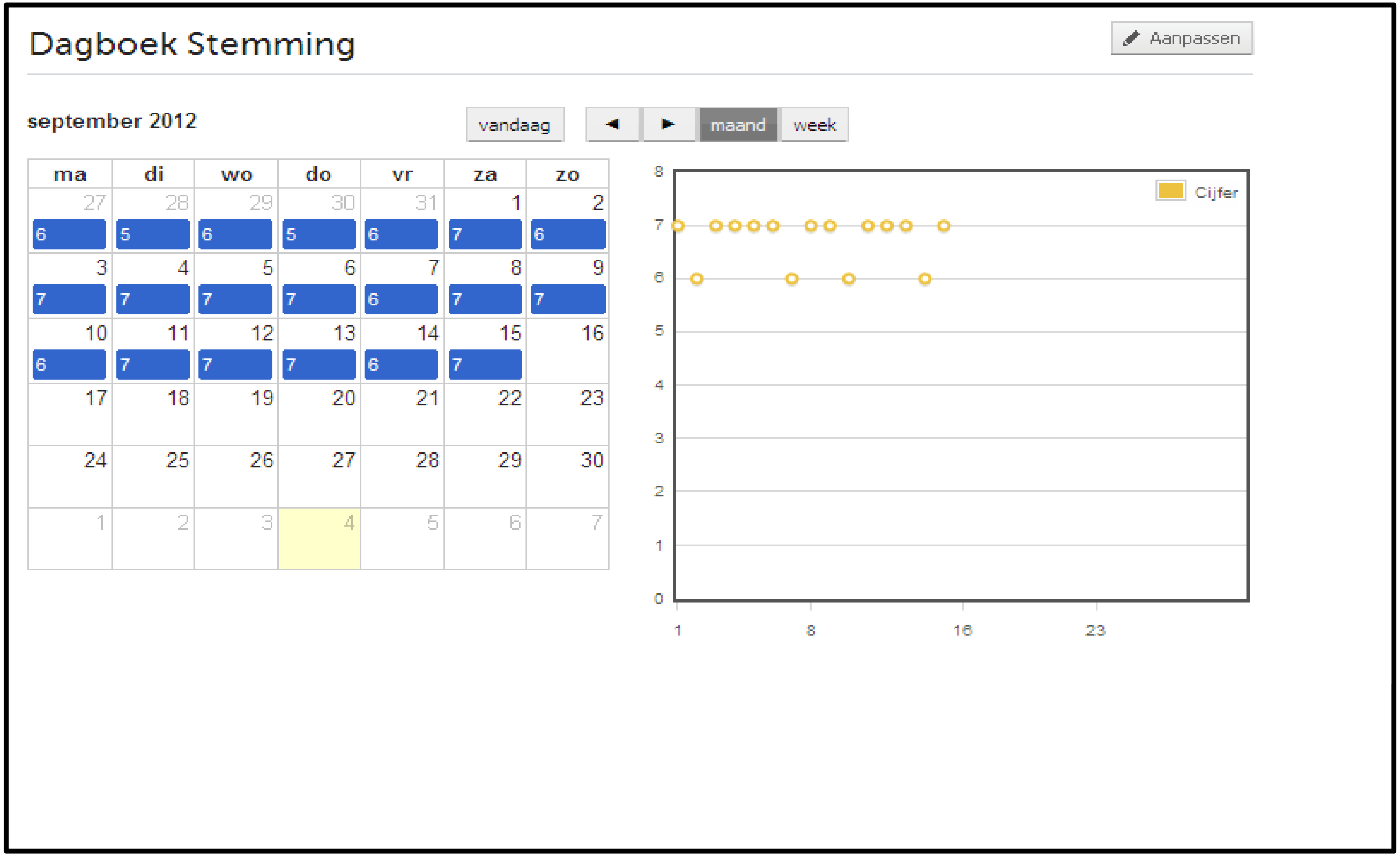
Dagboek Stemming
Aanpassen
september 2012
vandaag
maand
week
ma di wo do vr za zo
27 28 29 30 31 1 2
6 5 6 5 6 7 6
3 4 5 6 7 8 9
7 7 7 7 6 7 7
10 11 12 13 14 15 16
6 7 7 7 6 7
17 18 19 20 21 22 23
24 25 26 27 28 29 30
1 2 3 4 5 6 7
Cijfer
0 1 2 3 4 5 6 7 8
1 8 16 23

Dutch-English Glossary

- Aanpassen: edit
- Begeleid door: accompanied by
- Behandeling: treatment
- Beschikbaar: available
- Conversaties: conversations
- Dagboek: diary
- Les: lesson
- Maand: month
- Oefening: assignment
- Probleem: problem
- Stemming: mood
- Takens: tasks
- Vandaag: today
- Week: week
- Zorg: worry
- Zorgverleners: caregivers

Corresponding author: Anna S. Geraedts, MSc: a.s.geraedts@vu.nl
