# Supplementary material for: Long-Term Results of a Web-Based Guided Self-Help Intervention for Employees With Depressive Symptoms: Randomized Controlled Trial
Source: J Med Internet Res. 2014 Jul 9;16(7):e168. doi: 10.2196/jmir.3539 (PMC4115257; doi:10.2196/jmir.3539)
Supplement: Supplementary file 2 [file jmir_v16i7e168_app2.pdf]

CONSORT-EHEALTH Checklist V1.6.2 Report

(based on CONSORT-EHEALTH V1.6), available at [http://tinyurl.com/consort-ehealth-v1-6].

3539

Date completed
5/13/2014 11:26:12
by
Anna Geraedts

Long-term results of a Web-based guided self-help intervention for employees with depressive symptoms: randomized controlled trial

TITLE

1a-i) Identify the mode of delivery in the title
'Long-term results of a Web-based guided self-help intervention for employees with depressive symptoms: randomized controlled trial"

1a-ii) Non-web-based components or important co-interventions in title
no non-web-based components

1a-iii) Primary condition or target group in the title
'Long-term results of a Web-based guided self-help intervention for employees with depressive symptoms: randomized controlled trial"

ABSTRACT

1b-i) Key features/functionalities/components of the intervention and comparator in the METHODS section of the ABSTRACT
'The intervention consisted of problem solving treatment and cognitive therapy and contained 6 lessons. Participants were asked to submit weekly assignments via the website after completion of a lesson and subsequently they received feedback from a coach via the website."

1b-ii) Level of human involvement in the METHODS section of the ABSTRACT
'Participants were asked to submit weekly assignments via the website after completion of a lesson and subsequently they received feedback from a coach via the website."

1b-iii) Open vs. closed, web-based (self-assessment) vs. face-to-face assessments in the METHODS section of the ABSTRACT
'We recruited employees from 6 companies via the company's intranet and by putting up posters."

1b-iv) RESULTS section in abstract must contain use data
'A total of 231 employees were randomized to either the intervention group (n=116) or to care-as-usual (n=115). Completion of assessments varied between 54-74%. "

1b-v) CONCLUSIONS/DISCUSSION in abstract for negative trials
'This study showed that a worker-directed Web-based guided self-help intervention was not more effective than care-as-usual in reducing depressive symptoms among employees with depressive symptoms who were not on sick-leave over the period of one year."

INTRODUCTION

2a-i) Problem and the type of system/solution
'The Organisation for Economic Co-operation and Development (OECD) [4] has recently recommended to increase the evidence for worker-directed treatments of mental health problems and have highlighted the importance of intervening before employees take sick-leave. Early intervention (before sick-leave) is important because it may prevent worsening of mental health problems and consequently has the potential to reduce the costs of work absenteeism and loss of work productivity [4, 25, 26]. "

2a-ii) Scientific background, rationale: What is known about the (type of) system
'Research on the treatment of depression has been extensive and has shown that depression can be treated effectively with different forms of psychotherapies [13-18]. Traditionally, most types of psychotherapies are delivered face-to-face in mental health care settings, but there is increasing evidence for the effectiveness of treatments that are delivered via the Internet [19-22]. In general, studies on the effectiveness of Web-based interventions for the treatment of depressive symptoms show positive short-term effects [21], but there are fewer studies available which have also studied the long-term effects of Web-based interventions [21]. "

METHODS

3a) CONSORT: Description of trial design (such as parallel, factorial) including allocation ratio
'It is of importance to examine whether the improvement in both groups is sustainable over time or if there will be an increase of depressive symptoms in one or both groups. Therefore in this study we examined between group differences over a 1-year follow-up period on depressive symptoms, burnout symptoms, work performance, and anxiety symptoms. In addition we studied the effects of the intervention on absenteeism and we performed several subgroup analyses regarding; educational level, age, gender, working hours, and baseline depression score because different effects for these subgroups might be possible. "

3b) CONSORT: Important changes to methods after trial commencement (such as eligibility criteria), with reasons
no changes

3b-i) Bug fixes, Downtimes, Content Changes
'There were no changes to the content, bugs, or periods with downtime during the trial."

4a) CONSORT: Eligibility criteria for participants
'Employees with elevated depressive symptoms as measured by a score of 16 or higher on the Center for Epidemiologic Studies Depression scale [(CES-D) who were not on sick-leave were eligible to take part in the study. Furthermore, access to the Internet and an e-mail address were required. Participants were excluded if they had been using medication for depressive symptoms for less than one month or if they had a legal labor dispute with the employer. "

4a-i) Computer / Internet literacy
'Furthermore, access to the Internet and an e-mail address were required. "

4a-ii) Open vs. closed, web-based vs. face-to-face assessments:
'The design and short-term outcomes of this study have been described in detail elsewhere [39, 40]. We will therefore describe the design briefly. Participants were recruited via six different companies in the Netherlands; two banking companies, two research institutes, one security company, and one university through banners and digital pamphlets on the company's intranet and via posters. "

4a-iii) Information giving during recruitment

4b) CONSORT: Settings and locations where the data were collected
'Participants completed online questionnaires at baseline, post-treatment (8 weeks, t1), 6 months (t2), and 12 months (t3). "

4b-i) Report if outcomes were (self-)assessed through online questionnaires
'Participants completed online questionnaires at baseline, post-treatment (8 weeks, t1), 6 months (t2), and 12 months (t3). "

4b-ii) Report how institutional affiliations are displayed

5) CONSORT: Describe the interventions for each group with sufficient details to allow replication, including how and when they were actually administered

5-i) Mention names, credential, affiliations of the developers, sponsors, and owners

'The intervention Happy@Work [41] is a brief Web-based intervention delivered with minimal guidance. "

5-ii) Describe the history/development process

5-iii) Revisions and updating

'There were no changes to the content, bugs, or periods with downtime during the trial. "

5-iv) Quality assurance methods

5-v) Ensure replicability by publishing the source code, and/or providing screenshots/screen-capture video, and/or providing flowcharts of the algorithms used

'Screenshots of the intervention can be found in Figure 2 and in the Multimedia Appendix 1 [Multimedia Appendix 1]."

5-vi) Digital preservation

'The intervention Happy@Work [41] is a brief Web-based intervention delivered with minimal guidance. "
citation 41 is url

5-vii) Access

'At the start of the intervention, an account was generated by the researchers on the website and a coach was assigned to the participant on the website. Once the account was generated, an automatic e-mail was sent to the participant with a link to activate the account. "

5-viii) Mode of delivery, features/functionalities/components of the intervention and comparator, and the theoretical framework

'The themes are related to PST, CT or work related problems. "
referral to paper with more detailed information

5-ix) Describe use parameters

'Participants receive feedback on assignments from a coach. "

5-x) Clarify the level of human involvement

'Participants receive feedback on assignments from a coach. Coaches were trained Master level students in clinical psychology. All coaches used a protocol-treatment manual. To ensure treatment fidelity, all feedback was reviewed by a supervisor (AG) before it was placed on the website. Happy@Work is a tunnelled intervention which means that participants can start with a new lesson after they have received feedback on their assignments from a coach. "

5-xi) Report any prompts/reminders used

'Participants receive feedback on assignments from a coach. Coaches were trained Master level students in clinical psychology. All coaches used a protocol-treatment manual. To ensure treatment fidelity, all feedback was reviewed by a supervisor (AG) before it was placed on the website. Happy@Work is a tunnelled intervention which means that participants can start with a new lesson after they have received feedback on their assignments from a coach.
At the start of the intervention, an account was generated by the researchers on the website and a coach was assigned to the participant on the website. Once the account was generated, an automatic e-mail was sent to the participant with a link to activate the account. Participants used their e-mail address and a self-created password to log in once the account was activated. E-mail reminders were sent to participants when deadlines were not met. "

5-xii) Describe any co-interventions (incl. training/support)

'. Participants in both conditions were free to seek any additional (mental) health care. "

6a) CONSORT: Completely defined pre-specified primary and secondary outcome measures, including how and when they were assessed

'Participants completed online questionnaires at baseline, post-treatment (8 weeks, t1), 6 months (t2), and 12 months (t3). "
"Depressive symptoms
The primary outcome was depressive symptoms as measured by the Center for Epidemiological Studies Depression scale (CES-D) [46]. This questionnaire is widely used for identifying people with depressive symptoms. Its validity has been tested in different populations [47-49]. The CES-D consists of 20 items and the total score varies between 0 and 60. The Cronbach's alpha in this study was .82. A score of 16 or higher represents a clinically significant level of depressive symptoms [46]. The cut-off score of 16 was used in this study as an inclusion criterion.
Burnout symptoms
Burnout symptoms were measured with the Dutch version of the Maslach Burnout Inventory-General Scale (MBI) [50, 51]. This self-report questionnaire contains 15 items and three dimensions: emotional Exhaustion (5 items); Cynicism (4 items); reduced Professional Efficacy (6 items). Every item was scored on a 7-point Likert scale (0-6). Following the manual of the questionnaire [51], a total score for every dimension was calculated by adding the item scores and by dividing the total score by the number of items, with higher scores indicating more severe symptoms. We rescored the Professional Efficacy dimension with higher scores indicating less feeling of professional efficacy. The Cronbach's alpha's for the different dimensions in this study were .83 for Exhaustion, .83 for Cynicism, and .79 for reduced Professional Efficacy.
Work absenteeism
Work absenteeism was measured with the second part of the Trimbos and iMTA Questionnaire on Costs Associated with Psychiatric Illness (TiC-P) the Short Form Health and Labor Questionnaire (SF-HLQ) [52]. The participant was asked to report the total number of days absent from work because of illness in the time period between the two assessments; 8 weeks, 4 months, and 6 months. The recall period at baseline assessment was 3 months. Research has shown that participants can report valid and accurate rates of work absenteeism up to six months [53].
Work performance
We used the general work performance scale of the WHO Health and Work Performance Questionnaire (HPQ) [54] which contains four items. Item 4 gives the best and easiest indication of the participant's perception of their own work performance [55] by asking participants to rate their overall work performance during the past four weeks when compared to employees in comparable functions. We only report on that item in this study. Work performance was scored on a 7-point Likert scale with a higher score indicating poorer work performance compared to other employees [55].
Anxiety symptoms
The anxiety subscale of the Hospital Anxiety and Depression Scale (HADS) was used to measure anxiety symptoms [56]. The anxiety subscale of the HADS consists of 7 items. Scores range from 0 to 21, with higher scores indicating more anxiety. The HADS has shown good homogeneity and reliability in different normal and clinical Dutch samples [57]. The Cronbach's alpha in this study was .76.
Other measures
We included several demographic questions, working hours, and working days in the baseline questionnaire. "

6a-i) Online questionnaires: describe if they were validated for online use and apply CHERRIES items to describe how the questionnaires were designed/deployed

6a-ii) Describe whether and how "use" (including intensity of use/dosage) was defined/measured/monitored

described in more detail in other paper. we refer to this paper in the manuscript:
"The design and short-term outcomes of this study have been described in detail elsewhere [39, 40]. "

6a-iii) Describe whether, how, and when qualitative feedback from participants was obtained
described in more detail in other paper. we refer to this paper in the manuscript:
"The design and short-term outcomes of this study have been described in detail elsewhere [39, 40]. "
6b) CONSORT: Any changes to trial outcomes after the trial commenced, with reasons
no changes.
7a) CONSORT: How sample size was determined
7a-i) Describe whether and how expected attrition was taken into account when calculating the sample size
'The sample size was determined at 200 participants, based on a power of 0.80, an alpha of 0.05, and an expected drop-out percentage of 30% to show an effect-size Cohen's d of 0.50. "
7b) CONSORT: When applicable, explanation of any interim analyses and stopping guidelines
not applicable.
8a) CONSORT: Method used to generate the random allocation sequence
'An independent researcher made the allocation schedule with a computerized random number generator and the investigators had no knowledge of the schedule. The participants were informed about randomization outcome via e-mail. "
8b) CONSORT: Type of randomisation; details of any restriction (such as blocking and block size)
'An independent researcher made the allocation schedule with a computerized random number generator and the investigators had no knowledge of the schedule. The participants were informed about randomization outcome via e-mail. "
9) CONSORT: Mechanism used to implement the random allocation sequence (such as sequentially numbered containers), describing any steps taken to conceal the sequence until interventions were assigned
'An independent researcher made the allocation schedule with a computerized random number generator and the investigators had no knowledge of the schedule. The participants were informed about randomization outcome via e-mail. "
10) CONSORT: Who generated the random allocation sequence, who enrolled participants, and who assigned participants to interventions
'An independent researcher made the allocation schedule with a computerized random number generator and the investigators had no knowledge of the schedule. The participants were informed about randomization outcome via e-mail. "
11a) CONSORT: Blinding - If done, who was blinded after assignment to interventions (for example, participants, care providers, those assessing outcomes) and how
11a-i) Specify who was blinded, and who wasn't
participants and researchers were not blinded (not possible)
"The participants were informed about randomization outcome via e-mail."
11a-ii) Discuss e.g., whether participants knew which intervention was the "intervention of interest" and which one was the "comparator"
'This study was a randomized controlled trial with two arms: a Web-based guided self-help intervention, called Happy@Work, and a CAU group. "
"The participants were informed about randomization outcome via e-mail."
11b) CONSORT: If relevant, description of the similarity of interventions
not similar
12a) CONSORT: Statistical methods used to compare groups for primary and secondary outcomes
'Baseline differences in demographic variables and outcome measures were investigated using Chi-square tests and independent-sample t-tests. "
12a-i) Imputation techniques to deal with attrition / missing values
'All analyses were performed according to the intention-to-treat principle (ITT). Missing data were handled by multiple imputation via data augmentation. Data augmentation is an iterative Markov Chain Monte Carlo method to generate the imputed values assuming a multivariate normal distribution. Five imputations were used in all analyses and reported in the effectiveness analyses. "
12b) CONSORT: Methods for additional analyses, such as subgroup analyses and adjusted analyses
'We performed several a priori subgroup analyses on the primary outcome depressive symptoms. These subgroup analyses were: educational level, age (age <35 versus age ≥35), gender, working full time (≥36 hours per week) versus part time (< 36 hours per week), and high baseline score as defined by a score of ≥27 on the CES-D which is used more often as an indicator of more severe depressive symptoms [59-61]. In the subgroup analyses the specific subgroup was selected from all study participants and the difference between the groups over time was then compared. Furthermore, we performed a per-protocol analysis based on treatment completers (completed ≥3 lessons of the intervention).
Sensitivity analyses
We also performed all analyses on the data for 100 imputations. Since there is a current debate whether it is necessary to perform multiple imputations in combination with mixed-model analyses in longitudinal studies [62], we also performed the LMM analyses without multiple imputations. "

RESULTS

13a) CONSORT: For each group, the numbers of participants who were randomly assigned, received intended treatment, and were analysed for the primary outcome
CONSORT flowdiagram included
13b) CONSORT: For each group, losses and exclusions after randomisation, together with reasons
CONSORT flowdiagram included
"Of the 116 participants randomized to the intervention group, 9.5% (11/116) did not start or complete the first lesson of Happy@Work. A total of 67 participants (57.8%) were seen as treatment completers as they completed three or more lessons of the intervention. A total of 29 of 116 participants dropped out of the intervention on own request or due to long-during inactivity on the website. The other participants were not able to complete more lessons within the time limit of 7 weeks. The majority of the participants who dropped out did not report a reason for drop out. When reasons were reported (14/116) they pertained mostly to lack of time. "
13b-i) Attrition diagram
'Of the 116 participants randomized to the intervention group, 9.5% (11/116) did not start or complete the first lesson of Happy@Work. A total of 67 participants (57.8%) were seen as treatment completers as they completed three or more lessons of the intervention. A total of 29 of 116 participants dropped out of the intervention on own request or due to long-during inactivity on the website. The other participants were not able to complete more lessons within the time limit of 7 weeks. The majority of the participants who dropped out did not report a reason for drop out. When reasons were reported (14/116) they pertained mostly to lack of time. "
14a) CONSORT: Dates defining the periods of recruitment and follow-up
described in more detail in other paper
14a-i) Indicate if critical "secular events" fell into the study period
none.
14b) CONSORT: Why the trial ended or was stopped (early)
ended as planned.
15) CONSORT: A table showing baseline demographic and clinical characteristics for each group
table 1.
15-i) Report demographics associated with digital divide issues

table 1.

16a) CONSORT: For each group, number of participants (denominator) included in each analysis and whether the analysis was by original assigned groups

16-i) Report multiple “denominators” and provide definitions

reported in text and tables.

16-ii) Primary analysis should be intent-to-treat

yes, ITT used.

17a) CONSORT: For each primary and secondary outcome, results for each group, and the estimated effect size and its precision (such as 95% confidence interval)

yes, reported in text and tables

17a-i) Presentation of process outcomes such as metrics of use and intensity of use

'Of the 116 participants randomized to the intervention group, 9.5% (11/116) did not start or complete the first lesson of Happy@Work. A total of 67 participants (57.8%) were seen as treatment completers as they completed three or more lessons of the intervention. A total of 29 of 116 participants dropped out of the intervention on own request or due to long-during inactivity on the website. The other participants were not able to complete more lessons within the time limit of 7 weeks. The majority of the participants who dropped out did not report a reason for drop out. When reasons were reported (14/116) they pertained mostly to lack of time. ''
process evaluation will be reported separately.

17b) CONSORT: For binary outcomes, presentation of both absolute and relative effect sizes is recommended

not reported.

18) CONSORT: Results of any other analyses performed, including subgroup analyses and adjusted analyses, distinguishing pre-specified from exploratory

'Subgroup analyses
Data from the a priori subgroup analyses are reported in Table 4. There were no significant differences in depressive symptoms between the groups over time in any of the subgroups. Since the coefficients from the different subgroups were not substantially different from each other, there were no additional interaction effects tested to study whether there was a difference between the different subgroups over time. ''
''Sensitivity analyses
The analyses from the datasets without imputations and with 100 imputations did not reveal any relevant differences compared to the outcomes from the dataset with 5 imputations (data not shown). ''

18-i) Subgroup analysis of comparing only users

'Per protocol analyses
The per protocol analyses, in which the group of treatment completers was compared to the CAU group, did not reveal any significant results on the primary outcome depressive symptoms (unadjusted regression coefficient: -.48, 95%CI: -4.28 to 3.33, P = .80) and all secondary outcomes. The overall estimated mean difference for the MBI exhaustion dimension was .10 (95%CI: -0.24 to 0.43, P = .57), for the MBI cynicism dimension it was .14 (95% CI: -0.21 to 0.49, P = .42), for the MBI reduced professional efficacy dimension it was -.03 (95%CI: -0.48 to 0.41, P = .88), for work performance it was -.14 (95%CI: -0.79 to 0.51, P = .65), for absenteeism it was -1.66 (95%CI: -7.10 to 3.78, P = .54), and for anxiety symptoms it was .08 (95%CI: -1.06 to 1.23, P = .89). ''

19) CONSORT: All important harms or unintended effects in each group

no harms.

19-i) Include privacy breaches, technical problems

19-ii) Include qualitative feedback from participants or observations from staff/researchers

DISCUSSION

20) CONSORT: Trial limitations, addressing sources of potential bias, imprecision, multiplicity of analyses

20-i) Typical limitations in ehealth trials

'This study has several limitations. The first has to do with the attrition rate and handling of missing data. We were confronted with a high attrition rate which is more often seen in Web-based interventions [68, 69]. The attrition rates in this study were equal or lower compared to several similar studies on guided Web-based interventions for depression with long term follow-up assessments [64, 70, 71]. The bias that still may have been introduced was accounted for by applying multiple imputation techniques. Due to the current debate on the necessity of multiple imputations in combination with mixed-model analysis in longitudinal studies [62] we also performed mixed-model analysis without multiple imputations. The results were comparable, indicating that data were robust and multiple imputations might not have been needed. Second, the participants in this study were mainly Dutch white-collar workers with a high educational level. It is therefore uncertain whether the results can be generalized to the general working population or employees with a lower educational level. Although our subgroup analysis on educational level did not show significant differences, the subgroup analyses had a lack of power and only 36.4% of the study population had a low or middle educational level. Finally, as stated above, adherence to the intervention was low and only 57.8% completed at least 3 lessons of the intervention. Therefore, the analyses of comparisons between the intervention group and the CAU group compared the effects of a low adherence intervention, with many participants who only followed a small part of the intervention. The per protocol analyses did not show significant differences either, but had a lack of power as the analyses were only based on 42.2% of the intervention group. ''

21) CONSORT: Generalisability (external validity, applicability) of the trial findings

21-i) Generalizability to other populations

'Second, the participants in this study were mainly Dutch white-collar workers with a high educational level. It is therefore uncertain whether the results can be generalized to the general working population or employees with a lower educational level. Although our subgroup analysis on educational level did not show significant differences, the subgroup analyses had a lack of power and only 36.4% of the study population had a low or middle educational level.''

21-ii) Discuss if there were elements in the RCT that would be different in a routine application setting

will be reported separately in a process evaluation

22) CONSORT: Interpretation consistent with results, balancing benefits and harms, and considering other relevant evidence

22-i) Restate study questions and summarize the answers suggested by the data, starting with primary outcomes and process outcomes (use)

'This study examined the long-term effects of a worker-directed Web-based guided self-help intervention on depressive symptoms, several work-related outcome measures, and anxiety symptoms, compared to care as usual in employees with depressive symptoms who were not on sick-leave. This study did not affirm evidence for the long-term effectiveness of the Web-based intervention compared to CAU for any of the outcome measures. Overall, participants improved substantially on the primary outcome depressive symptoms between baseline and post-treatment assessment and these improvements sustained over the period of one year. This was also true for the work-related outcomes burnout symptoms and work performance as participants improved between baseline and post-treatment with sustainable effects up to 12 months. Overall, participants further improved after post-treatment assessment on anxiety symptoms. No difference between the two conditions in the course of symptoms was found on any of the outcome measures, however. Furthermore, there were no significant mean differences between the groups on duration of absenteeism during the follow-up period. We were not able to identify any subgroups that benefited from the treatment compared to CAU. Participants with a relatively high or low score on depressive symptoms, male or female, age <35 or over 35, working part time or working full time, having a low, middle or high educational level, or who had completed treatment or not did not improve more than the CAU group with respect to their depressive symptoms. "

22-ii) Highlight unanswered new questions, suggest future research

'The results of this study implicate that the intervention Happy@Work is not more effective in reducing depressive symptoms than CAU over the period of one year. Overall, participants improved substantially between baseline and post-treatment assessment on depressive symptoms and these improvements sustained over time. Participants also improved on the secondary outcomes which sustained over time. The large improvements on depressive symptoms in the CAU group were also found in two studies with comparable target groups of non-sick listed employees [27, 63]. It could therefore be possible that spontaneous recovery of depressive symptoms is more likely in this specific target group. Observational research following non-sick listed depressed employees over time could provide more insight.

The process evaluation which was performed alongside this trial concluded that the intervention was feasible for further implementation. However, based on the results of this trial we do not recommend to directly implement Happy@Work into routine practice as it was not more effective than CAU over time. It could, however, be possible that the intervention, even though it is not effective from a clinical perspective, could be effective from an economical perspective (e.g. cost-effective). This needs further investigation. Further, more research is needed to examine the possibilities of using e-mental health in the workplace setting. This research should focus on the needs of employees with mental health problems, and on the ideal moment when intervention is really necessary. "

Other information

23) CONSORT: Registration number and name of trial registry

'Trial registration: Nederlands Trial Register (NTR): NTR2993; http://www.trialregister.nl/trialreg/admin/rctview.asp?TC=2993 (Archived by WebCite at http://www.webcitation.org/6PL9pFC0n)"

24) CONSORT: Where the full trial protocol can be accessed, if available

text contains a reference to the study protocol

25) CONSORT: Sources of funding and other support (such as supply of drugs), role of funders

'Acknowledgements and funding

This study is funded by Body@Work Research Center for Physical Activity, Work and Health, TNO VUMC, Amsterdam and the EMGO Institute for Health and Care Research, VU University Amsterdam and VU University Medical Center Amsterdam. "

X26-i) Comment on ethics committee approval

'The study was approved by the Medical Ethics Committee of the VU University Medical Center (registration number 2011/2) and registered in the Dutch Trial Register (NTR2993). "

x26-ii) Outline informed consent procedures

described in more detail in other paper

X26-iii) Safety and security procedures

X27-i) State the relation of the study team towards the system being evaluated

'Conflicts of interest

The authors declare that they have no competing interests. "
